# Predictive Value of White Blood Cells, Neutrophils, Platelets, Platelet to Lymphocyte and Neutrophil to Lymphocyte Ratios in Patients with Acute Aortic Dissection

**DOI:** 10.21470/1678-9741-2020-0144

**Published:** 2020

**Authors:** Hong Liu, Dongze Li, Yu Jia, Rui Zeng

**Affiliations:** 1 Department of Emergency Medicine and Laboratory of Emergency Medicine, West China Hospital, and Disaster Medical Center, Sichuan University, Chengdu, China. E-mail: dongze.li@ymail.com; 2 Department of Cardiology, West China Hospital, Sichuan University, Chengdu, China.

Dear Editor,

We read with great interest the article “Association of Platelet to Lymphocyte and Neutrophil to Lymphocyte Ratios with In-Hospital Mortality in Patients with Type A Acute Aortic Dissection”, recently published in the *Brazilian Journal of Cardiovascular Surgery*^[1]^. This study included 96 type A acute aortic dissections (AAD) and indicated platelet to lymphocyte ratio (PLR) and neutrophil to lymphocyte ratio (NLR) as two novel indexes to predict in-hospital mortality. Type A AAD is a life-threatening disease, with a mortality of 50% in the first 48 hours, if not operated. Despite the progress in surgical techniques, in-hospital mortality can reach 25%^[2]^. It is known that the inflammation pathways involved in both Stanford type A and type B AAD and biomarkers based on inflammation could be potential risk stratification factors, including white blood cells, neutrophils and C-reactive protein. In the same way as the study by Selvi et al., our previous study also indicated that PLR was associated with in-hospital mortality in type B AAD^[3]^. However, there were no researches to compare the predictive values among the biomarkers. Therefore, we conducted a study to evaluate the predictive values of the biomarkers.

In this retrospective study, we enrolled patients diagnosed with AAD by computed tomography angiography (CTA) between January 2013 and September 2015 at West China Hospital, Sichuan University. Patients were excluded if they had active or chronic inflammatory disease, hepatopathy or chronic kidney disease, known malignancy, and hypersplenism. For each patient, baseline clinical characteristics, biochemical and hematological values, and the medical history were obtained at admission. The study was approved by the local ethics committee and all patients gave informed consent. Categorical data were presented as frequencies and percentages, and continuous data were presented as mean and standard deviation (SD) or median and interquartile range. A receiver operating characteristic curve (ROC) analysis was performed to investigate the diagnostic performance. A *P*-value <0.05 was considered statistically significant.

**Fig. 1:**
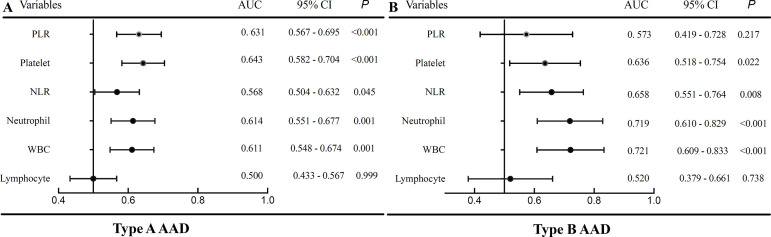
Area under the receiver operating characteristic curve of WBC, neutrophils, lymphocytes, platelets, NLR, PLR in patients with type A AAD (A) and type B AAD (B). AUC=area under the curve; CI=confidence interval; AAD=acute aortic dissection; WBC=white blood cells; NLR=neutrophil to lymphocyte ratio; PLR=platelet to lymphocyte ratio

The demographic and clinical characteristics of the study population were presented in Table 1. A total of 833 patients with AAD were enrolled (n=342 with type A AAD, n=491 with type B AAD). The overall in-hospital mortality was 15.7% (131/833), with 31.0% for type A AAD (106/342) and 5.1% for type B AAD (25/491). The ROC analysis showed white blood cell (WBC), neutrophil and platelet counts, NLR, and PLR could predict in-hospital mortality in patients with AAD (*P*<0.05, respectively) expect PLR in type B AAD (area under the curve [AUC]: 0.573, 95% confidence interval [CI] 0.419-0.728, *P*=0.217) (Figure 1). The AUC of in-hospital mortality for neutrophil and WBC counts and NLR did not present significant difference with each other in type A and type B AAD (*P*>0.1, respectively). The AUC of platelet and PLR did not show significant difference in type A AAD (0.643 *vs*. 0.631, *P*=0.707).

**Table 1:** Baseline clinical characteristics of patients with AAD.

Variables	Type A AAD (n=342)	Type B AAD (n=491)
Age, years	49.0±12.6	52.6±12.7
Males, n (%)	309 (90.4)	425 (86.6)
Smoking, n (%)	173 (50.6)	250 (50.9)
Hypertension, n (%)	193 (56.4)	384 (78.2)
WBC, *10^9^/L	11.8±4.1	10.3±4.1
Neutrophils, *10^9^/L	9.6±4.5	8.3±3.9
Lymphocytes, *10^9^/L	1.3±0.7	1.2±0.6
Platelets, *10^9^/L	174.3±88.1	197.4±90.7
NLR	7.7 (4.5, 11.9)	6.7 (3.9, 11.1)
PLR	128.0 (94.5,200.8)	164.6 (113.8, 247.0)
In-hospital death, n (%)	106 (31.0)	25 (5.1)

AAD=acute aortic dissection; NLR=neutrophil to lymphocyte ratio; PLR=platelet to lymphocyte ratio; WBC=white blood cells

In the present study, we found that WBC, neutrophil and platelet counts, and NLR were useful predictors of in-hospital mortality for patients with AAD and WBC, neutrophil and platelet counts might the better predictive biomarkers than PLR and NLR.

The increased neutrophil count and decreased lymphocyte count during inflammation led to the increased NLR, accepted as a convenient and cheap inflammatory marker. WBC, neutrophil and lymphocyte counts were all independent predictors of mortality in patient with acute myocardial infarction^[4]^. Apart from lymphocyte count, it was not possible to predict the in-hospital mortality in type A and type B AAD in the present study; there was no evidence for the predictive value of lymphocyte count. Therefore, we refer to WBC and neutrophil counts as the superior biomarkers for in-hospital mortality and the comparison of AUC confirmed the assumption. PLR was an emerging biomarker with the combination of hemostatic and inflammatory pathways, which was associated with major adverse cardiovascular events^[5]^. Previous studies have reported the relationship between low platelet count, PLR and in-hospital mortality type A AAD^[1,6]^. A U-shaped association between PLR and in-hospital death was observed in type B AAD^[3]^, and the AUC was 0.573 (95% CI 0.419-0.728, *P*=0.217). Therefore, there was insufficient evidence to indicate a better predictive value.

In conclusion, WBC, neutrophil and platelet counts, NLR and PLR have been associated with in-hospital mortality in patients with AAD. Neutrophil and platelet counts may be the most powerful biomarkers. Further exploration of the relationship between biomarkers and in-hospital mortality is warranted.
